# Data-driven policy making: a key step for the healthcare system: Angele Malatre

**DOI:** 10.1093/eurpub/ckac129.691

**Published:** 2022-10-25

**Authors:** A Malatre, E Ros, L Millet

**Affiliations:** Institut Montaigne, Paris, France

## Abstract

The work presented in this workshop suggest important room for improvement in data-driven health policy making in France and in Germany. We identify data generated by healthcare systems can be mobilized to inform four different aspects of public health and health care policy making:

1. Supporting patient-centered evaluation of healthcare services: micro-level data collection is key to developing evaluation tools which can be used to improve quality of care and patient experience, as evidenced by the development of PROMS and PREMS.

2. Adapting care supply to population needs: meso-level data collection enables local and national policy makers to design and implement healthcare programs and investments which fit population needs.

3. Developing targeted prevention policies: meso-level data collection can also be used to identify health hazards, improve population safety and limit health impact of exposure to sanitary and environmental risks, allowing local and national policy makers to articulate prevention strategies.

4. Informing healthcare research: patient and meso-level data are invaluable resources for health and life-sciences research, supporting identification of biomarkers and development of diagnostic tools and treatment.

Additionally, we identify that all four of these aspects of data-driven policy have implications at a local, national, and European level. In light of this, Institut Montaigne strongly advocates for a population-based approach, as implemented in Canada, relying on multiple datasets as well as individual and collective responsibility. We also stress the need for coordination at a European level, aware the current implementation of the European Health Data Space is an opportunity to leverage information from EU-wide databases. Data-driven public health and health care policy is a tool of public value and its use is critical to ensuring resilience of current health systems and addressing future crises.

